# Alternative career pathways for international medical graduates towards job market integration: a literature review

**DOI:** 10.5116/ijme.606a.e83d

**Published:** 2021-04-09

**Authors:** Tanvir C. Turin, Nashit Chowdhury, Mark Ekpekurede, Deidre Lake, Mohammad Lasker, Mary O'Brien, Suzanne Goopy

**Affiliations:** 1 Department of Family Medicine, Department of Community Health Sciences, Cumming School of Medicine, University of Calgary, Calgary, AB, Canada; 2 Alberta International Medical Graduates Association, Calgary, AB, Canada; 3 School of Languages, Linguistics, Literatures and Culture, University of Calgary, Calgary, AB, Canada; 4 Faculty of Nursing, University of Calgary, Calgary, AB, Canada

**Keywords:** Immigrants, alternative career pathways, transferable skills, international medical graduates, integration

## Abstract

**Objectives:**

To inform the current level of research about alternative career pathways for international medical graduates and synthesize knowledge of the barriers, facilitators and potential outcomes of alternative career pathways for international medical graduates.

**Methods:**

We searched MEDLINE, EMBASE, Scopus, and Google Scholar for relevant publications to February 2020. From 809 articles, after two levels of screening, 23 articles were selected. We conducted thematic content analysis and reported the findings of the study following the Preferred Reporting Items for Systematic Reviews and Meta-Analyses Extension for Scoping Reviews guidelines.

**Results:**

All 23 articles reported on alternative careers for international medical graduates in either the United States or Canada. A variety of study methods were noted, including original research, scoping reviews, reports for organizations, and commentaries. Studies incorporated a variety of methods, including surveys, focus groups, interviews, analysis of administrative documents, and program evaluation. Many potential benefits of alternative careers were reported for both international medical graduates and stakeholders. Barriers to pursuing alternative careers included both individual- and systemic-level barriers. Facilitators included skill-building workshops, targeted bridging programs, connecting with employers, supporting organizations, and international medical graduates.

**Conclusions:**

The scarce literature on alternative career pathways indicates that this research is beneficial for both international medical graduates and their host countries. Initiating capacity building programs for alternative career pathways for international medical graduates might be a worthy investment for host countries, especially in underserved areas. Pilot initiatives incorporating bridging programs for international medical graduates are recommended.

## Introduction

In this era of global migration, developed countries admit a substantial number of new immigrants every year through their immigration and refugee policies.^1^ These immigrant or newcomer populations come from a number of ethno-geographic backgrounds and exhibit differences in their socio-cultural and life practices that influence their settlement needs, including social care, education, health, and access to the labour market.^2^^-^^4^ Internationally trained medical professionals, also known as international medical graduates (IMGs)^5^ are a highly skilled but struggling segment of the immigrant population. IMGs are graduates of medical schools located outside the country where they intend to integrate professionally.^6^^,^^7^ They are also referred to as foreign medical graduates (FMGs), overseas trained graduates (OTGs), internationally trained physicians (ITPs), or internationally educated physicians (IEPs).^7^

The natural career course of these skilled professionals is to become a practicing physician; however, according to a Canadian report, as few as 6% of them will succeed in becoming a licenced physician.^8^ This extremely low rate of success is mostly attributable to systemic barriers, including the limited number of residency spots (a requirement for licencing) and discrimination against IMGs, as well as individual barriers such as socio-cultural and linguistic differences.^5^^,^^9^ Failing to integrate professionally as a practicing physician, many IMGs waste their skills by performing survival jobs^10^ completely unrelated to their educational background. In doing so, these highly skilled immigrant health professionals have become a symbol of the deskilling of highly skilled migrants in Canada.^11^

Against this backdrop, alternative careers, those jobs where IMGs' health-related knowledge, skills, and experience can be used, have emerged as a way to facilitate better professional integration for IMGs.^12^ However, during our preliminary exploration of the literature, we noticed the scarcity of literature in this area. Further, limited information is available regarding the potential outcomes of alternative career pathways (ACPs) for IMGs, the challenges they face, and how to facilitate their pursuit of alternative career options. Moreover, studies indicate there is a lack of awareness among IMGs and other stakeholders, such as potential employers of alternative jobs, the health- and wellness-related medical education sector, the immigrant-serving public, and private and non-profit bodies regarding alternative careers as a potential pathway to the professional integration of IMGs into the Canadian labor market.^12^

Through this rapid scoping review, we plan to explore the current level of international research regarding ACPs for IMGs and to synthesize the knowledge of the barriers and facilitators and potential outcomes of pursuing ACPs for IMGs. This understanding will help us determine and undertake the next steps in working on this important but overlooked issue.

## Methods

### Rapid review

In general, a rapid review is a form of review that synthesizes knowledge within a brief period, in which stages of a full-blown traditional review, such as a systematic scoping review, are simplified/fast-tracked and/or skipped to generate a quick outcome.^13^ A rapid review approach is usually selected for new or emerging research topics and updates of previous reviews and to quickly generate an outline of the evidence-based knowledge of a policy or practice for decision-makers. As professional integration of IMGs through ACPs is a relatively new and emerging topic of interest, and a quick overview of the relevant knowledge in the literature may help direct appropriate next steps, we adopted a rapid scoping review approach for this study.

Unlike standard systematic or scoping reviews, there is no established framework to follow in conducting a rapid review. Many studies have followed their own strategies or modified or adapted various proposed frameworks to meet their specific research goals.^14^^,^^15^ We conducted our rapid review by following a simplified framework for scoping reviews as employed by few other studies.^14^^-^^16^ All the stages of the scoping review were followed but a limited number of databases were used. As a Preferred Reporting Items for Systematic Reviews and Meta-Analyses (PRISMA) framework has not been developed for rapid scoping reviews, we followed the PRISMA-Extension for Scoping Reviews (PRISMA-ScR) checklist and guidelines (please see Appendix 1).^17^

### Stage 1: Identifying the research question

Identifying a clear and appropriate research question that limits the focus on the literature within a specific area of interest without excluding possible outcome variables is the first step of a scoping review. For this rapid scoping review, we identified the following research questions:

1.      What research has been undertaken regarding the professional integration of IMGs through ACPs?

2.      What are the benefits to IMGs and stakeholders of integrating into ACPs?

3.      What are the barriers and facilitators faced by IMGs when opting to pursue ACPs?

### Stage 2: Identifying relevant studies

### Published literature

As a limited number of databases are used to extract information for a rapid review, the databases need to be most representative of the topic of the research question and field.^14^ After thorough consideration and discussion among the members of the research team, the three most appropriate academic databases were selected for this review. As the subject of IMGs is primarily related to healthcare, we selected MEDLINE (Ovid) and EMBASE. However, as professional integration often involves multiple disciplines, including social sciences, migration, human geography, and education, we included Scopus to capture those particular aspects of IMGs' integration to ACPs. We developed the search terms, and these terms were further reviewed and validated by an experienced librarian. A complete list of search terms is provided in Table 1. In addition, we also conducted single citation searches and used a pearl growing approach or citation mining by reviewing the reference lists of all selected papers and publications and included additional studies that fulfilled our inclusion criteria (Appendix 2 shows the search strategy for MEDLINE and EMBASE).

### Grey literature

To obtain information from unpublished or in-progress studies, we also searched the grey literature. Extracting publicly available information on ACPs for IMGs was also incorporated into the grey literature search. We selected Google Scholar for our search, as it is one of the most commonly used grey literature sources, and studies indexed in other databases are often accessible through Google Scholar.^18^^,^^19^ Moreover, Google Scholar is a useful search engine to identify the organizational repositories and relevant information on websites of key national, international, professional, immigrant-serving, community, and government and non-government organizations. Search terms are listed in Table 1.

**Table 1:** Search terms

IMG [MEDLINE and Embase] International adj2 medical adj2 graduate* OR Foreign Medical Graduates [MeSH] OR foreign adj2 medical adj2 graduate* OR internationally adj2 trained adj2 doctor* OR internationally adj2 trained adj2 physician* OR internationally adj2 trained adj2 medical graduate* OR internationally adj2 educated adj2 physician* OR internationally adj2 trained adj2 health professional OR internationally adj2 educated adj2 health professional OR foreign adj2 trained adj2 doctor* OR foreign adj2 trained adj2 physician* OR foreign adj2 trained adj2 medical graduate* OR foreign adj2 educated adj2 physician* OR foreign adj2 educated adj2 health professional OR overseas adj2 trained adj2 doctor*
Career [MEDLINE and Embase] (Career Choice [MeSH] OR profession* adj3 integrat* OR alternative adj3 career OR integrat*)
For Scopus (International medical graduate*) OR (foreign medical graduate*) OR (internationally trained doctor*) OR (internationally trained physician*) OR (internationally trained medical graduate*) OR (internationally educated physician*) OR (internationally trained health professional) OR (internationally educated health professional) OR (foreign trained doctor*) OR (foreign trained physician*) OR (foreign trained medical graduate*) OR (foreign educated physician*) OR (foreign educated health professional) OR (overseas trained doctor*) AND (Career Choice OR profession* integrat* OR alternative career OR integrat*)
For Google Scholar ((International medical graduate) OR (foreign medical graduate) OR (internationally trained doctor) OR (internationally trained physician) OR (internationally trained health professional) OR (internationally educated health professional) OR (overseas trained doctor)) AND (career OR profession OR integration)

### Stage 3: Study selection

To extract the relevant studies on professional integration of IMGs through ACPs we used specific definitions of terms and appropriate databases to ensure sufficient and relevant coverage. We followed the PICOS structure (Table 2) in selecting studies for this review. We did not restrict studies based on country of origin or date of publication, but we excluded all studies focusing on residency-related issues of IMGs. We included studies published in the English language only.

**Figure 1:**
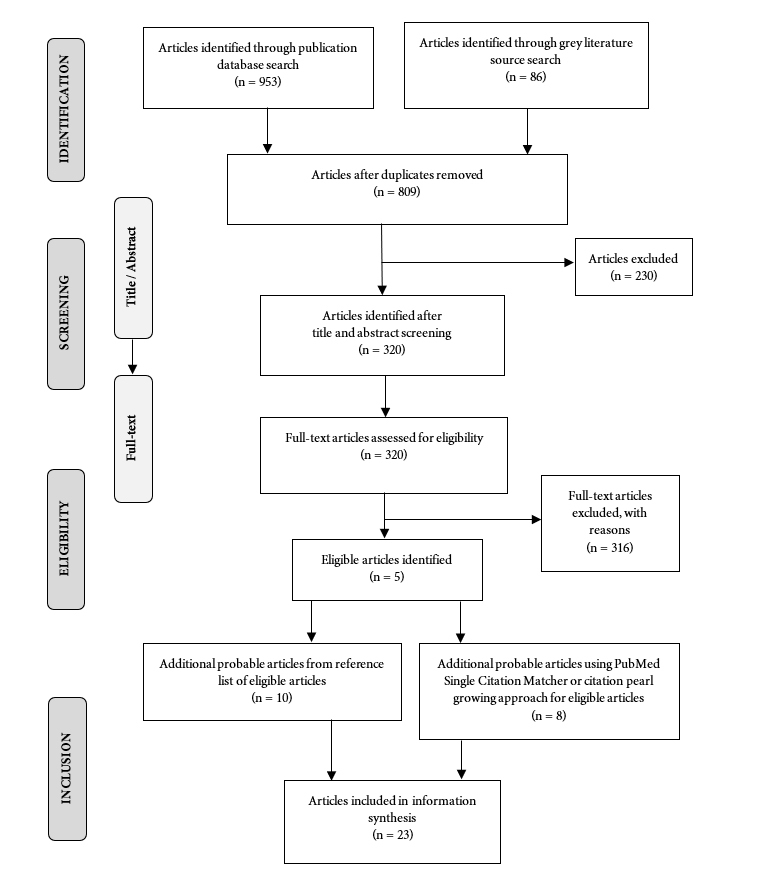
Study selection schematic

All search outcomes were assessed through a two-step screening process: (i) title-abstract review, and (ii) full-text review (Figure 1). Following common rapid review strategies,^14^ only one researcher conducted the screening. In the first screening step, the reviewer screened the papers based on the relevance of their titles and abstracts to our research questions. The full texts of the eligible abstracts were obtained and scrutinized for inclusion in the rapid review based on their relevance to alternative careers for IMGs. If the primary reviewer could not decide whether or not to include an article, other members of the research team reviewed it to achieve consensus.

**Table 2:** Inclusion and exclusion criteria

Inclusion criteria	Exclusion criteria
(P) Populations: Any study focusing on IMGs and the related stakeholders, including employers, and academics and community, system-level, and immigrant-serving organizations relevant to alternative career pathways suitable for IMGs (I) Interventions: Any approach, proposition, or assertion that facilitates or impedes the integration of IMGs through ACPs (C) Comparison: Studies compared, evaluated, assessed, or planned any alternative career choices for IMGs (O) Outcomes: Outcomes included but not limited to improved understanding of ACPs for IMGs or with the potential to influence the integration of IMGs into ACPs were included (S) Study design: Eligible study designs included but not limited to qualitative and quantitative studies, reviews, organizational reports, commentaries, letter to editors, and case studies No time restriction was given Studies published in English	Related to the professional integration of IMGs as physicians Discussed issues once IMGs in a residency program Studies discussed alternative careers for other health professionals (nurses, pharmacists, etc.) other than IMGs Studies not published in English

Note: ACP = alternative career pathway; IMG = international medical graduate.

### Stage 4: Charting the data

Data charting involved reading the full text of the eligible articles and identifying the key characteristics of those studies and their outcomes. The research team reviewer charted the information in a limited and predetermined data charting form. Study characteristics were extracted, including citation, study location, study type, study method, and study sample (Table 3). Further data on ACPs, barriers, facilitators, benefits of an alternative career choice, and take-away points were charted using emergent thematic coding (Table 4). EndNote (Clarivate Analytics, Philadelphia, Pennsylvania, USA) and Microsoft Excel (Microsoft Corporation, Redmond, Washington, USA) were used for data charting.

**Table 3:** Study characteristics

Study	Date	Location of study	Type of research	Methods	Sample/size
Alberta International Medical Graduate Association^27^	2019	Canada	Report	Workshops followed by surveys	22 IMGs who attempted to become licensed for three or more years and had failed were either unemployed or underemployed (i.e., working in a medical clinic or elsewhere and making <$20/h). Also, IMGs who were not interested in pursuing licensure or seeking an alternative career as a short-term goal were included.
American Academy of Physician Assistants^32^	1993	Florida, USA	Report	N/A	N/A
Anderson & Gilliss^41^	1998	USA	Report	N/A	N/A
Bhimji^31^	2010	Alberta, Canada	Commentary	N/A	N/A
Bhuiyan^23^		Ontario, Canada	Mixed method study	Surveys, interviews, and literature review and analysis of evaluation reports	A total of 67 in four cohort groups; 25 men and 42 women from over 24 different countries
Blain, Fortin & Alvarez^28^	2017	Canada and Quebec	Qualitative study	Semi-structured interviews	31 IMGs who hold degrees from outside the USA and Canada
Bourgeault, Neiterman, Lebrun, Viers & Winkup^20^	2010	Canada	Report	Interviews and document analysis	176 IEHPs from British Columbia, Manitoba, Ontario, and Quebec, including IMGs (67), IEN (70), and ITM (39)
Cawley^37^	1994	Maryland, USA	Letter to editor	N/A	N/A
Covell, Neiterman & Bourgeault^29^	2016	Canada	Qualitative study	Scoping review (Arksey & O'Malley^16^)	407 literature sources
Fasser & Smith^38^	1992	USA	Report	N/A	N/A
Flowers & Olenick^39^	2014	Florida, USA	Report	N/A	N/A
Fowkes, Cawley, Herlihy & Cuadrado^36^	1996	US	Report	N/A	N/A
Grossman & Jorda^40^	2006	Florida, USA	Report	N/A	N/A
Howard, Garman & McCann^26^	1995	USA	Quantitative study	A case-control study through objective evaluation measures through 3 tests: Test Item Bank, Objective Structured Clinical Examination, and Clinical Performance Examination	32 unlicensed IMGs who had graduated between 2-35 years earlier from medical schools in the Philippines, China, India, Cambodia, Mexico, Central America, South America, Poland, Hungary, or the Soviet Union; 6 recent graduates of the University of Southern California Physician Assistant Program as controls
Jablonski^22^	2016	Canada	Quantitative study	Cross-sectional design for capturing risk factors for the employment of IMGs; Cohort studies for the identification of risk factors for securing residency spots while utilizing the Access Centre	N=8,373 Non-English or French-speaking individuals
Jones^33^	2015	USA and Canada	Commentary	N/A	N/A
Liebich^34^	2007	Australia, but relevant to Canada	Report	N/A	N/A
Lim Consulting Associates^12^	2013	Canada	Report	Interviews	Series of interviews with occupational contacts (13 respondents), immigrant-serving organizations (12 respondents), and related organizations and internationally educated physicians (13 respondents)
Magnus^35^	2008	Ontario, Canada	Commentary	N/A	N/A
Ministry of Health and Long-Term Care^21^	2012	Ontario, Canada	Report	Interviews; focus groups; surveys; analysis of the administrative data	52 PAs (32 IMG-stream) 41 supervising physicians 33 administrative representatives, including Clinical Director/Manager/Other Administrative, Medical Administrator, CNO/Professional Practice, CEO/Executive Director Hospital Team survey: 148 Hospital Team focus groups: 13 sites Patient/Client satisfaction surveys: N/R Hospital administrative data CHC Purkinje administrative data PEPA PA encounter reports Physician Supervision time analysis
Neiterman, Bourgeault & Covell^30^	2017	Canada	Qualitative study	Scoping review (Arksey & O'Malley^16^)	148 articles/reports
Smith & Fowkes^25^	1983	USA	Quantitative study	Surveys administered in person, by telephone, and by mail	736 unlicensed IMGs in California, USA
Wick^24^	2015	USA	Quantitative study	Descriptive statistics and chi-square analysis or Fisher exact test to summarize outcomes	All IMGs (total 39) who completed the program through 2013 were included in the study; graduation year was collapsed into two categories (1991-2005, n=18; and 2006-2013, n=18)

Note: CEO = chief executive officer; CHC = community health center; CNO = College of Nurses of Ontario; IEN = Internationally Educated Nurse; IMG = international medical graduate; ITN =Internationally Trained Nurse; NP= nurse practitioner; N/A = not applicable; PA = physician assistant; PEPA = Physician-Employed Physician Assistant; US = United States; USA = United States of America.

### Stage 5: Collating, summarizing, and reporting the results

The final stage of a scoping review combines the findings from all eligible articles to deliver an evidence-based response to the original research question. Data were collected,

synthesized, and presented using summary tables to inform the current state of evidence regarding the professional integration of IMGs through ACPs. The charted data were examined and compared to identify any patterns in the information on alternative careers for IMGs. The results of this process were further scrutinized to identify key themes. Table 4 details the key findings of each study pertaining to the professional integration of IMGs through ACPs. One reviewer (NC) completed the data analysis, but all authors reviewed the interpretation and reporting.

## Results

### Literature search overview

The systematic search of the three academic databases (MEDLINE, EMBASE, and Scopus) identified 953 articles, with an additional 86 articles found through searching the grey literature (Google Scholar). After removing duplicates, 809 articles were identified for the title and abstract screening. After reviewing the titles and abstracts, 320 articles were chosen for the full-text screening. The full-text screening revealed five eligible articles for the study. Through hand searching and snowballing the references of these articles, an additional 18 articles were identified. Finally, 23 studies were selected for this rapid scoping review (Figure 1).

### Study characteristics overview

#### Location

Nine of 23 studies were conducted in the United States. Eleven studies were conducted in Canada, while one study focused on both Canada and the United States. Only one study was conducted in Australia; however, it described an ACP for IMGs that was tested in Canada.

#### Type of research

Most articles (11 of 23) within our eligible studies constituted reports written for different organizations. Eight studies were original research, four of which were quantitative, three were qualitative, and the remaining one used a mixed-method research approach. Two of the qualitative studies were scoping reviews; one of them included other internationally trained medical professionals (e.g., nurses, pharmacists, etc.) and none of them were focused on the integration of IMGs through ACPs. Three articles were commentaries written by researchers in academic journals, and one was a letter to the editor.

#### Time-period

Among our 23 articles of interest, 13 were published within the last 10 years (2010-2019). The remaining articles were published earlier.

### Methods and objectives of the studies

The studies were very diverse in terms of their methodologies and objectives. Four of the original studies and organizational reports used surveys, four involved interviews, and one involved focus groups. Five studies analyzed data from different sources, including policy documents,^20^ administrative data,^21^^,^^22^ evaluation reports,^23^ and a graduation database^24^ of a potential ACP program for IMGs. These approaches were used to inquire about the potential of IMGs for an alternative career,^25^ evaluate IMGs preparedness for an ACP through tests,26 capture the outcomes of a pilot ACP program for IMGs,^21^ review a support program,^22^ pilot a bridging program,^23^ evaluate an accredited program for ACPs,^24^ assess workshops,^27^ or to identify factors associated with professional integration.^12^^,^^20^^,^^28^

Two scoping reviews attempted to find any gaps in the literature on professional integration of internationally trained health professionals.^29^^,^^30^ The remaining articles included reports, commentaries, and a 'letter to the editor'.^31^^-^^36^ The themes were related to experiences of IMGs working in ACPs^31^^,^^36^ and issues associated with the integration of IMGs through ACPs^20^^,^^32^^,^^37^^,^^38^ and described programs that enable IMGs to pursue an ACP.^39^^,^^40^

### Definition of alternative career

We only found one study that provided a working definition for alternative careers for skilled immigrants in general and not specifically IMGs. It defined alternative careers as "those that immigrants pursue other than but related to the regulatory profession in which they were originally trained, that make use of and relate to an immigrant's skills and experience."^12^

### Benefits of an alternative career

Several studies identified how alternative careers could be beneficial for both IMGs and stakeholders (Table 4). Stakeholders may include employers who have hiring capacity in those alternative careers, healthcare systems, governing bodies, and the community.

#### Benefits for IMGs

An alternative career provides IMGs who feel frustrated due to their loss of professional identity with an opportunity to regain a decent professional status and career satisfaction. ^25^^,^^27^ Studies that reported on Physician Assistant (PA) positions for IMGs as an alternative career found that IMGs are interested in considering the profession, as it offers them a permanent career change instead of engaging in a lengthy and expensive process^36^ and often not viable career^25^ through physician licensure.^36^ Some considered this an interim step toward licensure as a physician^36^ while gaining knowledge and experience of local healthcare systems,^31^ obtaining Canadian medical experience,^20^ and maintaining their clinical proficiency.^31^ For some IMGs who did not obtain their medical degree from an institution approved in the International Medical Education Directory, an alternative career was the only option to consider.^22^

A report of an American program that fast-tracks IMGs to nursing noted that IMGs in the program reported that US nursing encompasses impressive levels of practice, often similar to those that many IMGs used to practice in their home countries.^39^ Many IMGs consider this path as an opportunity to re-enter the healthcare system.^25^ According to another study, female IMGs often give up on pursuing licensing and find alternative employment as midwives, nurses, or in alternative medicine, as these career paths are comparatively more certain than the physician licensure process.^20^

#### Benefits for stakeholders

One study showed that IMGs allowed to work as PAs provided an immediately available pool of primary healthcare providers for underserved populations who also may have shared their languages and cultures.^36^ This was reiterated in other studies where the need for primary care providers with culturally diverse backgrounds was apparent, particularly in places with a high number of immigrant populations,^25^ in rural and minority populations,^38^ or in underserved areas irrespective of the population demography.^26^^,^^33^ One study showed that the majority of the IMG-PA graduates from truncated programs worked in primary care and one-third practiced in medically underserved areas.^24^ Moreover, patients were happy to see a provider from the same cultural and linguistic background.^24^

A pilot project in Ontario, Canada employed IMGs in PA roles, as there were limited residency spots for IMGs, and they had shown a comparable level of PA competencies as articulated by the Canadian Association of Physician Assistants.^34^ One study pointed out that the number of applicants to educational programs for health professionals was decreasing, thereby resulting in a shortage of primary care providers, and these issues could immediately be compensated for^31^ by using IMGs in PA roles or clinical/surgical assistant roles in primary care settings.^38^

IMGs in nursing roles after completing a fast-track nursing program were highly preferred and welcomed by recruiters for several reasons. Firstly, they had the education and training of a physician and nurse practitioner.^39^ Secondly, it is far more cost-effective to employ nurse practitioners than fully licensed physicians in primary care.^39^^,^^40^

### Barriers to ACPs

Across studies, a range of barriers faced by IMGs in their pursuit of alternative careers has been described. Some barriers arise at the individual level, while others are more systemic in nature. These barriers are discussed further below, and the results across the studies identified in this review are detailed in Table 4.

#### Individual-level barriers

#### IMGs' lack of interest in ACPs

Most of the IMGs in one study never considered looking for an alternative career to being a physician.^27^ Many contemplate switching their careers only after reaching a stumbling block in the pursuit of licensure.^28^ Studies also have shown that many IMGs are not interested in or committed to alternate professions and consider only those positions that may help them obtain Canadian clinical experience to facilitate their pursuit of physician licensure, e.g., PA or Clinical Assistant.^33^^,^^36^

**Table 4:** Barriers and facilitators encountered by IMGs in pursuing alternative careers

Study	Year	Benefits of the alternative career choice	Barriers	Facilitators	Recommendations
Alberta International Medical Graduate Association^27^	2019	N/R	Lack of professionals who agree to take IMGs for job shadowing or volunteering Lack of IMG's persistence in seeking employment	One-to-one coaching Developing professional profiles of the candidates Introducing candidates to the employers	Career transition programs tailored to IMG needs that identify, sharpen, and promote the transferable skills of IMGs and connect them with the employer can successfully integrate IMGs into the labor market through alternative careers and assist IMGs to make timely, informed choices regarding their future career
American Academy of Physician Assistants^32^	1993	N/R	Opposition by others, including PA associations and hospital associations	Completing a full, accredited PA program	Allowing IMGs to be licensed as a PA without a complete program was proved ineffective and was opposed by PA associations
Anderson & Gilliss^41^	1998	N/R	Lack of reading comprehension and command of the English language	N/R	PA programs have attracted unlicensed IMGs as applicants to their programs
Bhimji^31^	2010	For IMGs: To learn about the healthcare system and maintain some proficiency For stakeholders: Quick compensation of shortage of physicians	Inability to formulate a complete treatment plan and to communicate the plan to the patient to the Canadian standard Lack of adequate training, knowledge, and communication skills in the healthcare-related workplace safety practice Funding Limited leadership among regulators and health authorities	Sponsored by organizations like Medicentres Canada Setting up a system with a centralized application process and establishing a group of qualified assessors and funding raised from potential employers and partially from the government (proposed)	A program under the supervision of physicians for IMGs could work as an alternative career for IMGs and a steppingstone toward licensure while compensating for the physician shortage
Bhuiyan^23^	2018	For IMGs: An opportunity to utilize their skills to facilitate their integration into the healthcare workforce For stakeholders: Enhance the provincial and national economies and build individual capacity	N/R	N/R	Bridging programs for internationally trained doctors have the potential to create positive individual and societal impacts through capacity building for integration to the non-licensed healthcare sector in Canada
Blain, Fortin & Alvarez^28^	2017		N/R	Taking an alternative path right from the beginning or as soon as they encounter a stumbling block Returning to school is a common strategy	Taking alternative paths is a solitary journey and most take them without consulting formal resources
Bourgeault, Neiterman, Lebrun, Viers & Winkup^20^	2010	For IMGs: Getting Canadian medical experience Female IMGs give up licensing and find alternative employment (as midwives, nurses, or in alternative medicine) due to the uncertainty of the licensure process For stakeholders: N/R	No help available for IMGs to pursue alternate careers, they do so on their own No retraining programs for IMGs to utilize Professional networking and proactivity are often not enough to pursue an alternate career	Professional networking and proactivity Fate/luck	To establish bridging programs that would allow IMGs to practice in a health-related field
Cawley^37^	1994	N/R	Opposition by others, including PA associations and hospital associations	Completing a full, accredited PA program	Attempts to grant unlicensed IMGs the opportunity of becoming a PA without meeting standard requirements are often failed by legislative efforts IMGs unable to pass state medical licensing exams should not require a solution involving the PA profession
Covell, Neiterman & Bourgeault^23^	2016	N/R	Starting work in "survival" jobs causes difficulty for IEHPs to re-enter their desired career in Canada IEHPs' limited knowledge in navigating professional and other resources in the host country	N/R	To understand the importance of the development of research and policy to employ the unused human resources
Fasser & Smith^38^	1992	For IMGs: N/R For stakeholders: Diminishing numbers of applicants to health profession educational programs and primary care providers To address the healthcare needs of rural and minority groups of the population	Opposition of PA organizations against allowing unlicensed IMGs to become PAs based on a certain score in the medical licensing exams	Accredited course	The issues of utilization of IMGs and the risks associated with diminished control over preparation of healthcare professionals need to be clearly articulated and concerns of PA organizations addressed
Flowers & Olenick^39^	2014	For IMGs: US nursing is similar to how IMGs practiced medicine in their countries Impressive level of practice by US nurse practitioners Opportunity to re-enter the healthcare For stakeholders: Recruiters actively seek IMGs to hire, as they have had physician, nurse, and NP education and training NPs are far more cost effective than physicians for primary care	N/R	N/R	IMGs with a Bachelor's or Master's in Nursing can enhance patient access to primary care in rural and urban areas. Moreover, they are the perfect fit for the socio-culturally and linguistically diverse and underserved group of population.
Fowkes, Cawley, Herlihy & Cuadrado^36^	1996	For IMGs: A permanent career change due to lengthy and expensive physician licensure process An interim step toward licensure as physicians For stakeholders: Immediately available pool of potential healthcare providers for underserved populations that may share their languages and cultures	Possible lack of socialization knowledge for PA role and physician-PA relationship Lack of interest among IMGs to become a PA Lack of commitment of IMGs to build a career as a PA rather than using it as a steppingstone Language skills, behavioral expectations, cultural issues, or personal family needs Cost of developing exams IMGs' lack of English-language skills Inability to verify past medical education Fraud on applications claiming prior medical education Lack of recent practice	Preparatory programs appear to lessen the barriers to PA training	IMGs are not equivalent to PAs without specific training in accredited programs Using public funds to offer preparatory programs in order to add to the primary care workforce may not be the wisest move
Grossman & Jorda^40^	2006	For IMGs: By choice when medical licensing was not possible For stakeholders: Fulfills the shortage of RNs, especially from minority groups, and is cost-effective	English language skills Perception of their role and position in healthcare is a possible barrier	Supporting programs for English language skills Socialization course	Unemployed or underemployed IMGs can be a suitable source for meeting the demand of minority nurses in the US through a fast-track program
Howard, Garman & McCann^26^	1995	For IMGs: N/R For stakeholders: An urgent need for more medical practitioners in underserved areas	English as a second language Cultural and language differences may have affected patient communication and satisfaction IMGs have a lower standard of knowledge and medical skills than officially trained PAs	Further training on basic science knowledge, clinical skills, and awareness of the rights and restrictions of the PA profession	AAPA-recommended IMGs are required to take a 12-15-month accredited PA training program and subsequently pass the national PA board exam and TOEFL to be licensed as PAs
Jablonski^22^	2012	For IMGs: The only good option for those whose medical degree is not recognized in the International Medical Education Directory IMGs' choices For stakeholders: N/R	N/R	N/R	Most members do not use the resources of the Access Centre The very low success rate for alternative career path (0.4%)
Jones^33^	2015	For stakeholders: Using trained physicians in a PA role addresses immediate needs in healthcare	Very low 15.6% retention rate of IMGs as PAs IMGs without adequate training, cultural adaptation, and quality control in PA role	Providing structured educational and professional development programs for PA role	Without adequate formal education using IMGs in PA role is a waste of time, energy, and resources Four months of training followed by hours of evaluations could not adequately prepare IMGs in PA roles and culture
Liebich^34^	2007	For IMGs: N/R For stakeholders: Qualified IMGs could be a potential resource as in Ontario there were more qualified IMGs than medical residency spots. Their competencies are comparable to formally trained PAs.	Concerns raised around using IMGs as PAs	N/R	Idea of PAs is spreading outside the US quickly and IMGs can play a role in this
Lim Consulting Associates^12^	2013	For IMGs: Not able to obtain license For stakeholders: Use of IMGs' transferable skills	Lack of employer awareness of IEP abilities and experience as a barrier to finding employment Language ability, cultural competency, basic employment skills, unfamiliarity with job search and the labor market, lack of time Lack of motivation Lack of sustained funding Insufficient training and tools	Accurate pre-arrival information, counseling, training, and guidance Bridging programs Employer linkages Funding supports Identifying skills transferability and competency matching	Physicians/dentists appear to have limited alternative career options Alternative careers should be deemed as a parallel option to the career as a physician, not as an option for failure Partnerships between occupation-related bodies (e.g., regulatory and certification authorities and associations), employers, educational institutions, service providers, and government are required The same challenges that hold back IEPs from medical licensing prevent them to find alternative careers (e.g., language and communication skills)
Magnus^35^	2008	For IMGs: A second career option for IMGs For stakeholders: N/R	N/R	N/R	Using IMGs as PAs in the future could impact plans to develop a steady stream of suitably trained individuals through Canadian universities
Ministry of Health and Long-Term Care^21^	2012	N/R	Lack of regulation of the PA role Timely development/ advancement of medical directives Funding is a barrier to PA role at hospital	PAs work with multiple Supervising Physicians to enable them to work across multiple teams Clarity around when a PA could be called Improved communication	The impact of IMG-PAs was perceived positively from their Supervising Physicians, patients, IMGs themselves, and from analysis of administrative data Low percentage (41%) of IMG stream PAs' interest on continuing to work as a PA
Neiterman, Bourgeault & Covell^30^	2017	N/R	The recruiters of PA programs are often not welcoming to IMGs as they think IMGs are only choosing this training as a stepping stone	N/R	The literature on IMGs' integration to alternative careers are scarce. The existing studies are more focused on what should be done as opposed to what is being done.
Smith & Fowkes^25^	1983	For IMGs: An alternative when considering physician licensure in the US or California is not a viable possibility A way of employment and job satisfaction For stakeholders: Need for primary care with culturally diverse populations	Experiencing discrimination in non-physician professions both as IMGs and as racial minorities Many employers and others in the medical profession do not believe that IMGs working in non-physician health occupations are beneficial to healthcare in the US	N/R	Training and testing procedures are compulsory for certification of IMGs as PAs
Wick^24^	2015	For IMGs: N/R For stakeholders: IMGs can to contribute to the primary care workforce, as well as specialty practice	IMGs may find it difficult to be accepted by the rural residents It's unpredictable if the IMGs will remain in the PA profession	Thorough applicant screening process Ensuring the following requirements before recruiting: Minimum 4,000 hours of clinical experience Personal statement explaining their role as PA professional Supporting students with English as a second language	Properly developed truncated PA programs for IMGs can develop competent IMG-PAs with better performing capability than formally trained PAs The majority of IMGs work in primary care and about one-third in medically underserved areas, which was the ultimate purpose of such a program Patients appreciated being able to see a provider who shares their cultural and linguistic background

Note: AAPA = American Academy of Physician Assistants; CAPA = Canadian Association of Physician Assistants; IEHP = internationally educated health professional; IEP = internationally educated physician; IMG = International Medical Graduate; ISO = immigrant serving organization; N/R = not recorded; NP = Nurse Practitioner; PA = Physician Assistant; RN = Registered Nurse; TOEFL = Test of English as a Foreign Language.

#### Delayed start of alternative career search

One study indicated that waiting too long to start looking for an alternative career may negatively impact the ability of IMGs to find alternative employment.^28^ According to that study, IMGs who joined a fast-track nursing program that prepares IMGs to work as a nurse practitioner attempted to obtain US medical residency for at least five years before considering alternative options.^39^ Many IMGs arrive in the host countries at a later stage of their life, have family responsibilities, and are not in a position to consider alternative careers that require 'starting over'.^12^

#### Language ability and cultural competency

Studies have indicated that IMGs may fail in alternative career roles due to language differences and a lack of cultural competency,^36^^,^^40^ particularly in non-physician healthcare roles that require patient communication and satisfaction, such as PA and Nurse Practitioner.^26^^,^^31^^,^^41^

#### Unaware of the new work environment

It was argued by one study that IMGs working as PAs displayed a lack of knowledge of their new roles as a PA and the respective professional behaviour.^30^^,^^36^ Similar doubt about IMGs' perceptions of nurse practitioner roles and responsibilities was expressed in another study.^40^ Uncertainty regarding IMGs' lack of knowledge about work environments and professional attitudes may inhibit recruiters from employing IMGs in alternative professions.^12^

#### Financial barriers and job uncertainty

Some alternative careers require further academic upgrading for IMGs, which can often be quite extensive and expensive.^12^ Moreover, a lack of certainty of securing employment after the investment of money and time in a program for alternative careers inhibits IMGs from pursuing alternative careers, particularly those requiring academic upgrading and investment of time and money.^28^

### Systemic barriers

#### Lack of information in alternative career options

Immigrant IMGs may receive random pieces of information regarding alternative career options from immigrant-serving organizations (ISOs) that help skilled immigrants by providing professional information; however, there is often no structured source of information and resources available for them.^12^ Moreover, lack of knowledge about the labor market of the alternative careers, unfamiliarity with job search techniques, and lack of a professional network in the intended occupation impede the integration of IMGs into alternative careers.^12^

#### Lack of systematic support

One study reported that IMGs who found an alternative career had completed their professional journeys alone.^28^ They did not receive systematic support or consultation from experts. Their decisions were made on the basis of personal reflection and understanding the status of professional integration for IMGs in Canada.^28^

#### Lack of opportunities for employer engagement

Lack of local work experience for the respective jobs is an important barrier to IMGs' pursuit of alternative careers.^12^^,^^27^ A pilot workshop for IMGs bridging to alternative careers showed that managing a work placement, observership, internship, and volunteer opportunities is very difficult, without which securing a job is nearly impossible.^27^

#### Lack of sustained funding

A lack of sustained funding to ISOs that help skilled immigrants with their professional integration impedes the provision of their service.^12^ Funding is required to build partnerships with potential employers and other stakeholders and to develop bridging programs to prepare IMGs for the professions and connect them with potential employers.^12^

#### Not recognizing transferable skill sets

Recognizing IMGs' transferable skills is crucial to identifying and obtaining a suitable alternative career.^12^^,^^27^ In one study, IMGs reported that they had received extensive training in diagnosis and management of patients. Due to the specific nature of this training, employers and IMGs may not recognize the transferability of these skills to alternative careers.^12^ One respondent in that study suggested that IMGs' skills need to be systematically assessed in collaboration with potential recruiters and relevant experts.^12^

#### Discrimination to IMGs in ACPs

IMGs have reported facing discrimination due to working in non-physician professions, despite being trained as a physician, or being a member of a racial minority.^25^ Further, many employers believe IMGs working in a non-physician healthcare workforce is not beneficial to the overall healthcare system.^25^ They do not understand why an internationally qualified physician would seek employment in an alternate field, often one for which they are overqualified.^36^ Moreover, they are reluctant to hire IMGs, presuming that they are interested in using the position only as a stepping stone to their career goal.^30^

### Facilitators to ACPs

Table 4 summarizes the facilitators reported across the studies identified in this review. The various facilitators are discussed further below.

#### One-to-one consultation

One-to-one coaching or consultation sessions may be beneficial to inform and prepare IMGs for their professional integration through ACPs.^27^ Such consultation is essential to assess their prior education and work experiences, as well as to identify transferable skills and help them make informed career choices.^12^^,^^27^ In addition, providing IMGs with a few highly salient successful case stories may help IMGs in transitioning to an alternative career.^12^

#### Skill-building workshops

One study demonstrated that workshops to prepare IMGs for particular alternative careers are useful.^27^ Moreover, including potential employers in the workshops helped increase their awareness regarding the potential of alternative careers as a way to professionally integrate IMGs.^27^

#### Bridging programs with placements

Developing bridging programs that help determine IMGs' transferable skills and build relevant new skills are essential for successful integration to alternative careers.^27^ However, one study indicated that IMGs preferred part-time programs that allow them to work and earn a living while upgrading themselves for alternative careers.^12^ Effective bridging programs should offer IMGs the opportunity of job shadowing, mentoring, and internships, as those are useful for both employers and IMGs.^27^

#### Connecting IMGs with employers

A pilot project in Alberta, Canada attempted to connect IMGs with potential employers by forwarding employers structured profiles of IMGs.^27^ This approach facilitated their employment, and the employers showed their interest in receiving additional similar referrals.^27^ Another study found that both IMGs and potential employers need to be informed about the potential requirements and relevant transferable skills of IMGs to better understand each other's employment needs.^12^

#### Alternative career roadmap and tools

Key informants in one study reported that some organizations use their individual resources to help IMGs in their search for alternative careers.^12^ However, adopting a systematic approach and developing and applying a roadmap in collaboration with all stakeholders will make this more productive and practical.^12^ An assessment tool should be developed that takes into account prior education and experience and identifies gaps in the requirements for both employers and IMGs.^12^

## Discussion

In this rapid scoping review, we attempted to identify the current research and evidence-based understanding of alternative careers for IMGs. Although a few comprehensive studies were conducted, they indicate that ACPs are beneficial for both IMGs and the host country, especially where there is a good potential for utilizing IMGs' existing skillsets, such as non-physician health-related jobs. Studies showed that working as a physician is central to the individual identity of IMGs; however, when that goal becomes difficult to pursue due to the many barriers they encounter, they look for a career that is closely related to their profession, such as those in allied health and wellness professions.^42^ Despite the lower economic and professional outcomes from many of these careers, they opt for these alternatives as a way to remain in the health and wellness field.^42^ IMGs in health research, health informatics, and health management have the potential to enhance local and national economies by facilitating professional integration.^23^ Increasing the employability of skilled immigrants and creating capacities to contribute to society can also have a positive impact on both the individual IMGs and society.^8^ However, female IMGs may be more inclined to pursue alternative careers to establish their career more quickly, as the uncertainty of the physician licensure process undermines their family life.^20^

However, similar to the pathway to becoming a physician, IMGs are not spared from certain individual and systemic barriers in their pursuit of alternative careers. Individual barriers such as 'IMGs' lack of interest in ACPs', and 'Delayed start of alternative career search' indicate a lack of determination for alternative careers among some IMGs and a feeling of pushback toward the goal of becoming a practicing physician. 'Language ability and cultural competency' and 'Unaware of the new work environment' imply the need for socio-cultural and professional acculturation, respectively. 'Financial barriers and job uncertainty', on the other hand, demonstrates the vulnerability of these immigrant professionals and the struggles they face in the host country.

Reported systemic barriers such as 'Lack of information in alternative career options', 'Lack of systematic support', 'Lack of opportunities for employer engagement', and 'Lack of sustained funding' indicate the lack of systemic awareness and effort and action required to help IMGs integrate professionally through alternative careers. Other systemic barriers, namely 'Not recognizing transferable skill sets' and 'Discrimination to IMGs in ACPs', demonstrate the systemic reluctance and negative attitude toward IMGs' professional integration through alternative careers.

To find success in an alternative career, IMGs need to identify their hard and soft skills, knowledge, competencies (i.e., what they are good at) and aptitudes (i.e., what they can learn easily) required for the career.^27^^,^^43^ The studies in this review indicated that although IMGs have many transferable skills and aptitudes for learning, they generally need to refresh some of those skills, particularly within the context of the host country. They also require support and an opportunity to acquire new skills to be competitive candidates for those careers. 'One-to-one consultation', 'Skill-building workshops', and 'Bridging programs with placements' were proposed or piloted in some studies to facilitate this aspect of career development. Moreover, as an alternative career is a new field for the IMGs and there is a lack of awareness among employers about the potential of IMGs in alternative careers, helping IMGs to 'connect with employers' by supporting organizations through capacity development or professional development for IMGs/immigrants was found effective. Overall, 'an alternative career roadmap and tools' for both IMGs and the supporting organizations for alternative careers was urged.

The instrumental driver of an alternative career is being unable to obtain a position in the primary career.^27^^,^^44^ According to one study, only about a quarter (24.7%) skilled immigrants work in their original profession, whereas more than half (54.7%) of their Canadian-born counterparts work in the field for which they have been trained.^45^ Similar to IMGs, an alternative career is increasingly becoming a viable career pathway for the majority of other internationally trained health professionals, such as nurses, pharmacists, occupational therapists, midwives, medical laboratory technologists, and others^.20^

### Implications for medical education

Despite the potential of alternative careers, various factors, including being required to upgrade skills related to the alternative jobs, obtaining a professional license, obtaining certification or membership, and opportunities in the job market for those seeking alternative careers, are crucial. A pragmatic approach to alternative careers for IMGs through capacity building might be a worthy investment for host countries to help this under-appreciated group of immigrants contribute towards the health and wellness of the country, especially in underserved areas.^40^ Many studies reported that through meticulously developed bridging programs, IMGs could effectively be trained for different roles; however, these activities will benefit from proper engagement activities from their inception. Helping IMGs pursue ACPs instead of undertaking low-skilled jobs boosts IMGs' prospects of better integrating into the labor market and reduces the financial burden and economic loss caused by the underutilization and underemployment of skilled immigrants.^46^^,^^47^ Further, across the studies in our review, the IMG population was treated as a single homogeneous population, where in reality it is very heterogeneous. Factors such as cultural background, socioeconomic constraints in the new country, or time already spent in the new health system will be informative and need to be incorporated in our understanding of ACPs for IMGs.

### Strengths and limitations

This review has several strengths. The first is that it involved an extensive search strategy that included academic databases and grey literature. The conventional approach of conducting a rapid review is through a single database search; however, we searched three major academic databases with our research questions in mind. We also undertook a grey literature database search to help expand the scope of our search. However, this review also has some limitations we need to acknowledge. The inclusion criteria were quite broad; however, considering the fact that there was scarce research on ACPs of IMGs we deemed that as necessary to identify all available information on this topic. Further, in this review, we did not assess the quality of the literature, because of the diverse record types included in this review.

## Conclusions

Through this rapid scoping review, we determined that the literature is scarce in the relatively new area of IMGs' professional integration through ACPs. Nevertheless, the existing studies indicate that these pathways have the potential to benefit both IMGs and the host country. Moreover, there is a myriad of barriers that need to be addressed to facilitate these undertakings, particularly the promotion of targeted bridging programs and skill development workshops for IMGs to help them pursue alternative careers. Helping IMGs navigate their pursuit of ACPs and the many barriers they might face in achieving their goals will increase the economic and societal integration of IMGs. As a next step, engagement with potential employers is required to create awareness among them, as well as to guide integration from employers' perspectives. Academics, support providers, industry stakeholders, and policymakers need to be engaged in this work to ensure uptake and impact.

### Conflict of Interest

The authors declare that they have no conflict of interest.

## Supplementary material

Supplementary file 1 Appendix 1. Preferred Reporting Items for Systematic Reviews and Meta-Analyses Extension for Scoping Reviews (PRISMA-ScR) checklist

Supplementary file 2 Appendix 2. Full electronic search strategy for MEDLINE and EMBASE
