# Supplementary material for: Alternative career pathways for international medical graduates towards job market integration: a literature review
Source: Int J Med Educ. 2021 Apr 9;12:45–63. doi: 10.5116/ijme.606a.e83d (PMC8415394; doi:10.5116/ijme.606a.e83d)
Supplement: Supplementary file 2 — Appendix 2. Full electronic search strategy for MEDLINE and EMBASE [file ijme-12-45-S2.pdf]

Appendix 2.

Full electronic search strategy for MEDLINE and EMBASE

No.	Search term (and operator)	Results (MEDLINE)	Results (EMBASE)
1	exp Foreign Medical Graduates/	3433	247
2	Foreign medical graduate*.mp.	3556	742
3	(Foreign adj2 medical adj2 graduate*).mp. [mp=title, abstract, original title, name of substance word, subject heading word, floating sub-heading word, keyword heading word, organism supplementary concept word, protocol supplementary concept word, rare disease supplementary concept word, unique identifier, synonyms]	3597	825
4	(international adj2 medical adj2 graduate*).mp. [mp=title, abstract, original title, name of substance word, subject heading word, floating sub-heading word, keyword heading word, organism supplementary concept word, protocol supplementary concept word, rare disease supplementary concept word, unique identifier, synonyms]	894	1041
5	(internationally adj2 trained adj2 doctor*).mp. [mp=title, abstract, original title, name of substance word, subject heading word, floating sub-heading word, keyword heading word, organism supplementary concept word, protocol supplementary concept word, rare disease supplementary concept word, unique identifier, synonyms]	4	4
6	(internationally adj2 trained adj2 physician*).mp. [mp=title, abstract, original title, name of substance word, subject heading word, floating sub-heading word, keyword heading word, organism supplementary concept word, protocol supplementary concept word, rare disease supplementary concept word, unique identifier, synonyms]	8	7
7	(internationally adj2 trained adj2 medical graduate*).mp. [mp=title, abstract, original title, name of substance word, subject heading word, floating sub-heading word, keyword heading word, organism supplementary concept word, protocol supplementary concept word, rare disease supplementary concept word, unique identifier, synonyms]	2	2
8	(internationally adj2 educated adj2 physician*).mp. [mp=title, abstract, original title, name of substance word, subject heading word, floating sub-heading word, keyword heading word, organism supplementary concept word, protocol supplementary concept word, rare disease supplementary concept word, unique identifier, synonyms]	3	3
9	(internationally adj2 educated adj2 health adj2 professional*).mp. [mp=title, abstract, original title, name of substance word, subject heading word, floating sub-heading word, keyword heading word, organism supplementary concept word, protocol supplementary concept word, rare disease supplementary concept word, unique identifier, synonyms]	17	19
10	(internationally adj2 trained adj2 health adj2 professional*).mp. [mp=title, abstract, original title, name of substance word, subject heading word, floating sub-heading word, keyword heading word, organism supplementary concept word, protocol supplementary concept word, rare disease supplementary concept word, unique identifier, synonyms]	3	3
11	(foreign adj2 trained adj2 doctor*).mp. [mp=title, abstract, original title, name of substance word, subject heading word, floating sub-heading word, keyword heading word, organism supplementary concept word, protocol supplementary	23	27

	concept word, rare disease supplementary concept word, unique identifier, synonyms]		
12	(foreign adj2 trained adj2 physician*).mp. [mp=title, abstract, original title, name of substance word, subject heading word, floating sub-heading word, keyword heading word, organism supplementary concept word, protocol supplementary concept word, rare disease supplementary concept word, unique identifier, synonyms]	51	46
13	(foreign adj2 trained adj2 medical adj2 graduate*).mp. [mp=title, abstract, original title, name of substance word, subject heading word, floating sub-heading word, keyword heading word, organism supplementary concept word, protocol supplementary concept word, rare disease supplementary concept word, unique identifier, synonyms]	4	4
14	(foreign adj2 educated adj2 physician*).mp. [mp=title, abstract, original title, name of substance word, subject heading word, floating sub-heading word, keyword heading word, organism supplementary concept word, protocol supplementary concept word, rare disease supplementary concept word, unique identifier, synonyms]	6	5
15	(foreign adj2 educated adj2 health professional*).mp. [mp=title, abstract, original title, name of substance word, subject heading word, floating sub-heading word, keyword heading word, organism supplementary concept word, protocol supplementary concept word, rare disease supplementary concept word, unique identifier, synonyms]	2	2
16	(overseas adj2 trained adj2 doctor*).mp. [mp=title, abstract, original title, name of substance word, subject heading word, floating sub-heading word, keyword heading word, organism supplementary concept word, protocol supplementary concept word, rare disease supplementary concept word, unique identifier, synonyms]	49	59
17	(overseas adj2 trained adj2 health professional*).mp. [mp=title, abstract, original title, name of substance word, subject heading word, floating sub-heading word, keyword heading word, organism supplementary concept word, protocol supplementary concept word, rare disease supplementary concept word, unique identifier, synonyms]	6	5
18	1 or 2 or 3 or 4 or 5 or 6 or 7 or 8 or 9 or 10 or 11 or 12 or 13 or 14 or 15 or 16 or 17	4040	1884
19	(Foreign Medical Graduates or Foreign medical graduate* or (Foreign adj2 medical adj2 graduate*) or (international adj2 medical adj2 graduate*) or (internationally adj2 trained adj2 doctor*) or (internationally adj2 trained adj2 physician*) or (internationally adj2 trained adj2 medical graduate*) or (internationally adj2 educated adj2 physician*) or (internationally adj2 educated adj2 health adj2 professional*) or (internationally adj2 trained adj2 health adj2 professional*) or (foreign adj2 trained adj2 doctor*) or (foreign adj2 trained adj2 physician*) or (foreign adj2 trained adj2 medical adj2 graduate*) or (foreign adj2 educated adj2 physician*) or (foreign adj2 educated adj2 health professional*) or (overseas adj2 trained adj2 doctor*) or (overseas adj2 trained adj2 health professional*)).tw,kf.	1598	0
20	(Foreign Medical Graduates or Foreign medical graduate* or (Foreign adj2 medical adj2 graduate*) or (international adj2 medical adj2 graduate*) or (internationally adj2 trained adj2 doctor*) or (internationally adj2 trained adj2 physician*) or (internationally adj2 trained adj2 medical graduate*) or (internationally adj2 educated adj2 physician*) or (internationally adj2 educated adj2 health adj2 professional*) or (internationally adj2 trained adj2 health adj2 professional*) or (foreign adj2 trained adj2 doctor*) or (foreign adj2 trained adj2 physician*) or (foreign adj2 trained adj2 medical adj2 graduate*) or (foreign adj2 educated adj2 physician*) or (foreign adj2 educated adj2 health	1581	1704

	professional*) or (overseas adj2 trained adj2 doctor*) or (overseas adj2 trained adj2 health professional*)).ti,ab.		
21	exp Career Choice/ or career.mp.	55843	408593
22	(profession* adj3 integrat*).mp. [mp=title, abstract, original title, name of substance word, subject heading word, floating sub-heading word, keyword heading word, organism supplementary concept word, protocol supplementary concept word, rare disease supplementary concept word, unique identifier, synonyms]	1141	1521
23	(alternative adj3 career).mp. [mp=title, abstract, original title, name of substance word, subject heading word, floating sub-heading word, keyword heading word, organism supplementary concept word, protocol supplementary concept word, rare disease supplementary concept word, unique identifier, synonyms]	81	101
24	integrat*.mp.	508251	612683
25	21 or 22 or 23 or 24	562313	1002698
26	18 and 25	426	327
